# Long-term sialidase-specific immune responses after natural infection with cholera: Findings from a longitudinal cohort study in Bangladesh

**DOI:** 10.3389/fimmu.2022.1067737

**Published:** 2022-12-22

**Authors:** Fahima Chowdhury, Afroza Akter, Taufiqur Rahman Bhuiyan, Rajib Biswas, Md. Golam Firoj, Imam Tauheed, Jason B. Harris, Regina C. Larocque, Allen G. Ross, Nigel A. J. McMillan, Richelle C. Charles, Edward T. Ryan, Stephen B. Calderwood, Firdausi Qadri

**Affiliations:** ^1^ Infectious Diseases Division, icddr, b (International Centre for Diarrhoeal Disease Research, Bangladesh), Dhaka, Bangladesh; ^2^ Menzies Health Institute Queensland, Griffith University, Gold Coast, Australia; ^3^ Department of Biology, Xavier University of Louisiana, New Orleans, AK, United States; ^4^ Department of Pediatrics, Harvard Medical School, Boston, MA, United States; ^5^ Division of Infectious Diseases, Massachusetts General Hospital, Boston, MA, United States; ^6^ Department of Medicine, Harvard Medical School, Boston, MA, United States; ^7^ Rural Health Research Institute, Charles Sturt University, Orange, New South Wales, Australia; ^8^ Department of Immunology and Infectious Diseases, Harvard School of Public Health, Boston, MA, United States; ^9^ Department of Microbiology, Harvard Medical School, Boston, MA, United States

**Keywords:** sialidase, cholera, *V. cholerae*, immunity, nutrition

## Abstract

**Background:**

Immune responses that target sialidase occur following natural cholera and have been associated with protection against cholera. Sialidase is a neuraminidase that facilitates the binding of cholera toxin (CT) to intestinal epithelial cells. Despite this, little is known about age-related sialidase-specific immune responses and the impact of nutritional status and co-infection on sialidase-specific immunity.

**Methods:**

We enrolled 50 culture-confirmed *Vibrio cholerae* O1 cholera cases presenting to the icddr,b Dhaka hospital with moderate to severe dehydration. We evaluated antibody responses out to 18 months (day 540) following cholera. We assessed immune responses targeting sialidase, lipopolysaccharide (LPS), cholera toxin B subunit (CtxB), and vibriocidal responses. We also explored the association of sialidase-specific immune responses to nutritional parameters and parasitic co-infection of cases.

**Results:**

This longitudinal cohort study showed age-dependent differences in anti-sialidase immune response after natural cholera infection. Adult patients developed plasma anti-sialidase IgA and IgG responses after acute infection (P<0.05), which gradually decreased from day 30 on. In children, no significant anti-sialidase IgA, IgM, and IgG response was seen with the exception of a late IgG response at study day 540 (p=0.05 compared to adults). There was a correlation between anti-sialidase IgA with vibriocidal titers, as well as anti-sialidase IgA and IgG with anti-LPS and anti-CtxB antibody responses in adult patients, whereas in children, a significant positive correlation was seen only between anti-sialidase IgA and CtxB IgA responses. Stunted children showed significantly lower anti-sialidase IgA, IgG, and IgM antibody responses and higher LPS IgG and IgM antibody responses than healthy children. The anti-sialidase IgA and IgG responses were significantly higher in cases with concomitant parasitic infection.

**Conclusion:**

Our data suggest that cholera patients develop age-distinct systemic and mucosal immune responses against sialidase. The stunted children have a lower anti-sialidase antibody response which may be associated with gut enteropathy and the neuraminidase plays an important role in augmented immune response in cholera patients infected with parasites.

## Introduction

Cholera remains a major public health problem in resource-limited countries, and cholera outbreaks following natural and human-made disasters underscore the overall global health threat posed by cholera. Many epidemiological studies reveal that natural infection with *V. cholerae* provides long-term immunity; however, the correlation of protection afforded by an immune response to a range of *V. cholerae* antigens is not well understood (1). Currently available oral cholera vaccines (OCVs) provide around 65% protective efficacy (PE) for 3~5 years against cholera (2). Protective efficacy varies by age group, with protective efficacy in young children under 5 years of age approximating 40% with a short duration of protection (2, 3). Several studies in endemic settings demonstrate that vaccines and natural infection induce significant vibriocidal, cholera toxin B subunit (CtxB), toxin coregulated pilin (TcpA), and lipopolysaccharide/O-specific polysaccharide (LPS/OSP) antibody responses. Many of these immune responses fall within a few months of cholera, despite immunity afforded by previous infection lasting for years. To inform future possible vaccine development, we here describe an analysis of sialidase-specific antibodies following cholera, with analysis out to 18 months.

*Vibrio cholerae* O1 specific sialidase is a neuraminidase that facilitates the binding of CT to intestinal epithelial cells (4). Sialidases are enzymes that catalyze the cleavage of terminal sialic acids from oligosaccharides and glycoconjugates (5). *V.cholerae* sialidase has a unique essential Ca2+ ion and this sialidase can hydrolyze both 2,3- and 2,6-linked glycosidic bonds (6, 7). Sialidase plays an important role in cholera infection by removing sialic acid residues from higher-order gangliosides on gut epithelial cell membranes to generate monogangliosides (GM1), the binding receptor for CT (8), and by helping *V. cholerae* colonize the intestine in heavily sialylated areas like the intestinal epithelium (4).

We have recently identified and explored sialidase-specific immune responses in a number of our immune analyses in cholera patients. First, in an analysis of cloned plasmablasts in patients recovering from cholera, clones recognizing sialidase were the third most frequently identified, following clones recognizing *V. cholerae* OSP and CT (9). Second, we performed micro-array-based immune profiling of sera and antibody-in-lymphocyte supernatant (ALS- a marker of mucosal immune responses) in adult cholera patients and identified sialidase as a frequent target (1). Third, we analyzed short-term anti-sialidase immune responses out to 30 days following cholera in adults and children, finding that the most prominent immune responses occurred in adults, perhaps suggesting immune boosting from previous infection (4).

In our current study, we report the evolution of anti-sialidase immune responses in adults and children out to 18 months following cholera, and assess the correlation of anti-sialidase immune responses with other anti-*V. cholerae* immune responses, and assess the impact of nutritional and parasitic co-infection status on sialidase-specific immune responses.

## Methods

### Study participant and sample collection

The International Centre for Diarrhoeal Disease Research (icddr,b), Dhaka hospital serves more than 100,000 diarrhoeal patients including ~ 20,000 cholera patients annually, the majority of them living in or around Dhaka city. We enrolled participants from January 2018 to December 2020, who came to icddr,b Dhaka hospital with acute watery diarrhea (AWD), having signs of moderate to severe dehydration assessed by the World Health Organization (WHO) guidelines (10). Inclusion criteria included a stool culture positive for *V. cholerae* O1, aged 2 to 60 years old, and without any significant comorbid conditions.

On study day 1, we identified the patient based on inclusion and exclusion criteria and stool was sent for culture for *V. cholerae.* The patient was enrolled on study day 2 if stool culture was positive for *V. cholerae* O1. These individuals were followed up to day 540. The routine stool microscopy examination (RME) was also done for the enrolled patients. We aimed to compare the clinical features and immunological parameters between the children aged 2 to 17 years and adults aged 18 to 60 years for analysis. Blood samples were collected to measure different kinds of immunological markers such as antibodies to sialidase, LPS, CtxB, and vibriocidal titers on days 2, 7, 30, 90, 180, and 540 after enrollment of each index case.

### Microbiological analysis

Stool culture for *V.choleare* was done to confirm the cholera cases by taurocholate-tellurite-gelatin agar (TTGA) media. The serological confirmation for *V.choleare* colonies was done by overnight incubation of plates, through the slide agglutination method (11). Stool parasites including protozoa and helminths were inspected using the direct microscopic method for intestinal parasites after confirmation with *V.cholerae* in patients. In RME, we prepared two slides for each cholera patient. For the rice watery stool, a drop of stool was examined straightly below a cover slip whereas, for the semisolid/solid stools, we prepared a thin solution mixed with ~ 2gm of stool and normal saline. The 1% Lugol’s iodine was used to make the second slide. The 22 mm cover glasses were used for the slides, and the entire film was examined under light microscopy for the detection of parasites.

### Sialidase-specific IgA, IgG, and IgM antibodies in plasma

We measured anti-sialidase IgG, IgM, and IgA responses in plasma using standard enzyme-linked immunosorbent assay (ELISA) protocols. To determine anti-sialidase antibody titers, the ELISA plates were coated with sialidase (2.5 µg/mL) in carbonate buffer. Sialidase was prepared from *Vibrio cholerae* O1 strain N16961 that overexpressed from pXT7 cloning vector in *E. coli* as recombinant polyhistidine proteins (1). We added 100 µL of plasma to each well (diluted 1∶25 in 5% non-fat dry milk in phosphate-buffered saline-0.05%) and detected the presence of antigen-specific antibodies using horseradish peroxidase-conjugated goat anti-human IgG, IgM or IgA antibody (diluted 1∶1000 in 5% non-fat dry milk in phosphate buffered saline-0.05%Tween) (Southern Biotech, Birmingham, AL). After incubation at 37°C for 1 h and washing, we developed the plates with ortho-phenylenediamine (OPD; Sigma, St. Louis, MO) in 0.1 M sodium citrate buffer (pH 4.5) and 0.012% hydrogen peroxide (H_2_O_2_) and determined the optical density at 450 nm for 5 minutes at 30s intervals with an Eon Microplate Spectrophotometer (BioTek, CA). The rate of change in optical density was measured as milli-absorbance units per minute. The rate of change in optical density of each sample was then normalized and expressed as ELISA units by calculating the ratio of the sample to a standard of pooled sera (4, 12). The maximal rate of OD change was expressed in milli absorbance units per minute, and ELISA units were standardized by calculating the ratio of the test sample to a sample of pooled convalescent stage serum from patients who had recovered from cholera, run as a positive control on each plate.

### Vibriocidal antibody assay

We measured vibriocidal antibody responses in plasma by using guinea pig complement (Sigma-Aldrich Chemie GmbH) and *V. cholerae* O1 Ogawa (X-25049) and Inaba (T-19479) as the target organisms (10, 13). A variation of one log of the pooled sera is acceptable for vibriocidal assay over different days. Pooled convalescent stage serum from patients who had recovered from cholera, run as a positive control or standard on each plate for ensuring reproducibility. We defined the vibriocidal responses as the reciprocal of the highest dilution resulting in ≥50% reduction of the optical density compared to that of control wells without plasma. We considered those individuals as responders who showed a ≥4-fold increase in vibriocidal titer from baseline (14).

### LPS, and CtxB-specific IgA, IgG, and IgM antibodies in plasma

We assessed LPS, and CtxB-specific IgA, IgG, and IgM antibody responses in plasma by using an enzyme-linked immunosorbent assay (ELISA), as previously described (13, 15, 16). The homologous LPS was measured based on specific serotypes of *V.cholerae* O1 or Inaba. We added 100 µl of plasma (diluted 1:50 for LPS and 1:100 for CtxB in 0.1% bovine plasma albumin in phosphate-buffered saline-0.05% Tween) per well and detected responses using horseradish peroxidase-conjugated secondary antibodies to human IgG, IgA, or IgM (Jackson Immuno Research, West Grove, PA; 1:1,000 dilution). After incubation at 37°C and washing, we developed the plates with orthophenylene diamine (Sigma, St. Louis, MO) in 0.1 M sodium citrate buffer (pH 4.5) and 0.012% hydrogen peroxide followed by reading the plates kinetically at 450 nm for 5 min (17, 18). We considered individuals who showed a ≥2-fold increase in LPS, and CtxB responses compared to baseline values on study day 2 to be responders (14).

### Nutritional status

The nutritional status of children was measured using a z-score cut-off point according to the WHO Global Database on Child Growth and Malnutrition. The z-scores were calculated using the WHO Child Growth Standards for children aged between zero and 60 months or the WHO Growth References for school-aged children and adolescents. We used a z-score cut-off point of < −2 SD to classify low weight-for-age (underweight), low height-for-age (stunting) and low weight-for-height (wasted) (10).

### Statistical analyses

The demographic and clinical characteristics of cholera patients were recorded as numbers with percentage and median with inter-quartile range (IQR). The distribution of the nutritional status of the children and clinical characteristics in children and adult cases at different time points were analyzed with the Kruskal Wallis test with a 5% level of significance. The correlation between anti-sialidase responses and other clinical characteristics in both children and adult patients was analyzed using the Pearson correlation test at a 5% level of significance using t-test.

All the analyses were done using R statistical software. For visualization, the line graph, boxplot, and line graph with error bars were generated using “ggplot2” and “ggpubr” packages and for z score calculation “zscorer” package was used.

## Results

### Study population

**Figure 1:**
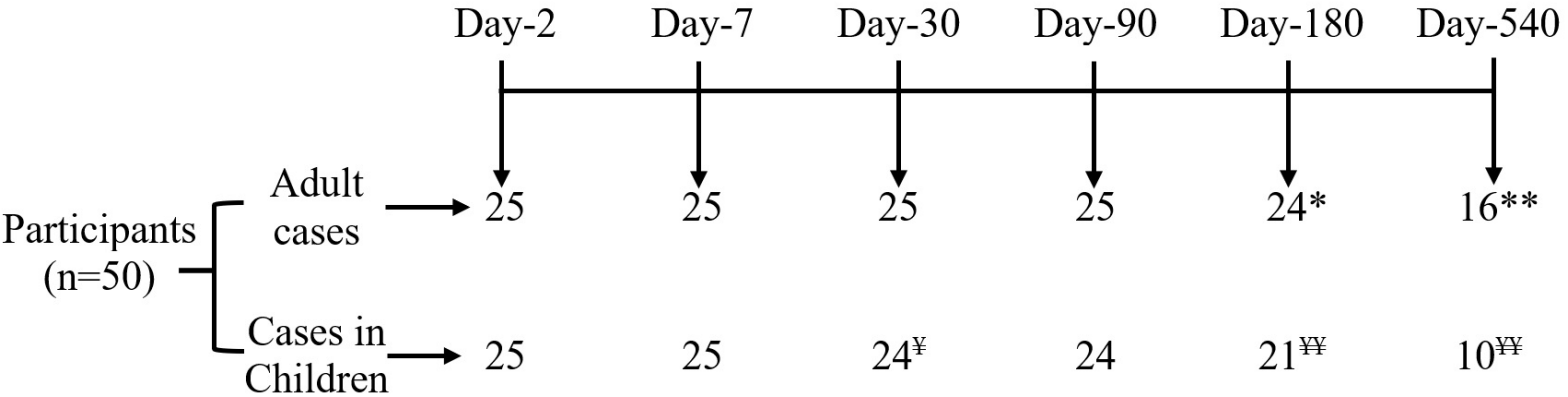
Consort diagram of participant disposition. *Refuse to continue study at day 180 (n=1) and **migration out at day 540 (n=8); ^¥^ Refuse to continue study at day 30 (n=1) and ^¥ ¥^ migration out on 180 (n=4), and 540 (n=10).

We enrolled 50 culture-confirmed cholera cases (25 adults and 25 children) with moderate to severe dehydration and the consort diagram of the study participants and their follow up at each study points have been presented in the **Figure 1**. Most of the cases were male (adult cases: 60% male; cases in children: 68% male). The median age of adult cases was 32 years (IQR: 27-40 years), and for cases in children 5 years (IQR: 3-8 years). Most of the cases had O-positive blood (48% for adults and 60% for children). Based on the WHO “Z Score” classification of children, 20% were stunted (height-for-age), 44% were underweight (weight-for-age) and 47% were wasted (weight-for-height) (**Table 1**).

**Table 1:** Demographic and anthropometric characteristics of the participants enrolled in the study.

Characteristics		Cholera Patients Adult N=25	Cholera Patients Children N=25
Gender	Male (%)	15 (60%)	17 (68%)
Age (years)	Median (IQR)	32 (27, 40)	5 (3, 8)
Blood group, no. (%)	O	12 (48%)	15 (60%)
	A	5 (20%)	4 (16%)
	B	7 (28%)	6 (24%)
	AB	1 (4%)	0 (0%)
Health Status for Child	Stunted (HAZ)	–	5 (20%)
	Underweight (WAZ)	–	11 (44%)
	Wasted (WHZ)	–	9 (47.4%)

WAZ, weight-for-age; WHZ, weight-for-height; HAZ, height-for-age, as described by World Health Organization anthropometric classifications (http://www.who.int/childgrowth/software/en/). Children with a Z score < −2 were categorized as moderately or severely undernourished for that category. Children with a Z score ≥ −2 were categorized as non-moderately or severely malnourished.

### Anti-sialidase antibody responses among cholera patients

**Figure 2:**
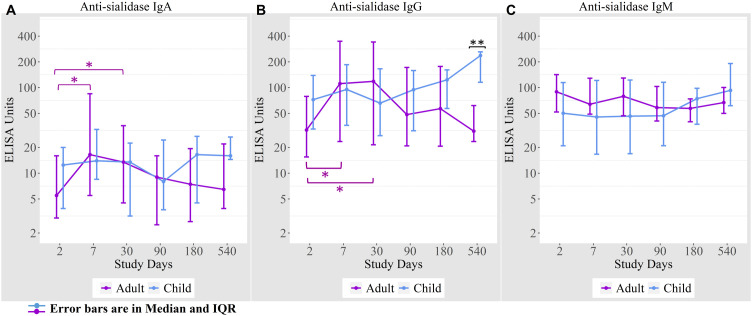
Anti-sialidase antibody responses in cholera cases in adults and children over the course of the study. **(A)** Anti-sialidase IgA responses among adults and children **(B)** Anti-sialidase IgG responses among adults and children **(C)** Anti-sialidase IgM responses among adults and children. Kruskal-Wallis test has done in order to identify the significant difference in median responses of anti-sialidase. *p=0.05- 0.01; **p=0.01-0.001. The purple asterisks denote comparisons in adult patients to day 2, and the black asterisks denote the difference between children and adult cases.

We measured anti-sialidase immune responses among adults and children suffering from cholera out to day 540 following infection (**Figure 2**). Adult patients developed significant increases in plasma anti-sialidase IgA and IgG responses on days 7 and day 30 following infection and these responses then gradually returned to baseline.

There was no the anti-sialidase IgA, IgG, and IgM significant response seen in children. From day 90 onwards, anti-sialidase IgA, IgG, and IgM immune responses increased compared to adults, but the differences were only significant for anti-sialidase IgG on day 540.

### Correlation between anti-sialidase responses and other antibody responses

Anti-sialidase IgA and IgG responses in adults were associated significantly with vibriocidal responses for both the Ogawa and Inaba serotypes; and with the antibody responses to CtxB and LPS for different isotype of antibodies. In children, a significant positive association was seen only between anti-sialidase IgA and anti-CtxB IgA responses (**Table 2**).

**Table 2:** Correlations matrix showing correlations between anti-sialidase responses and others immunological parameters.

		Adult Cases			Cases in Children		
		Anti-sialidase IgA	Anti-sialidase IgG	Anti-sialidase IgM	Anti-sialidase IgA	Anti-sialidase IgG	Anti-sialidase IgM
Vibriocidal Assay	Ogawa	0.3*	0	0.1	0.1	-0.1	-0.1
	Inaba	0.4*	0.1	0.2*	0.1	0	0.1
ELISA Assay	CtxB-IgA	0.5*	0.3*	0.2	0.3*	0.1	-0.1
	CtxB-IgG	0.3*	0.2*	0.1	0.1	-0.1	-0.1
	CtxB-IgM	0	-0.1	0.1	0	-0.1	0.1
	LPS-IgA	0.6*	0.3*	0	0.1	-0.1	0
	LPS-IgG	0.5*	0.2*	0	-0.1	-0.3*	-0.1
	LPS-IgM	0.2*	0	0	0	-0.2	0

Pearson correlation have calculated and tested with t-test for the significance relation at 5% level of significance.

* indicate significant correlation at the level of significance 0.05.

### Immunological responses among well-nourished versus malnourished children

**Figure 3:**
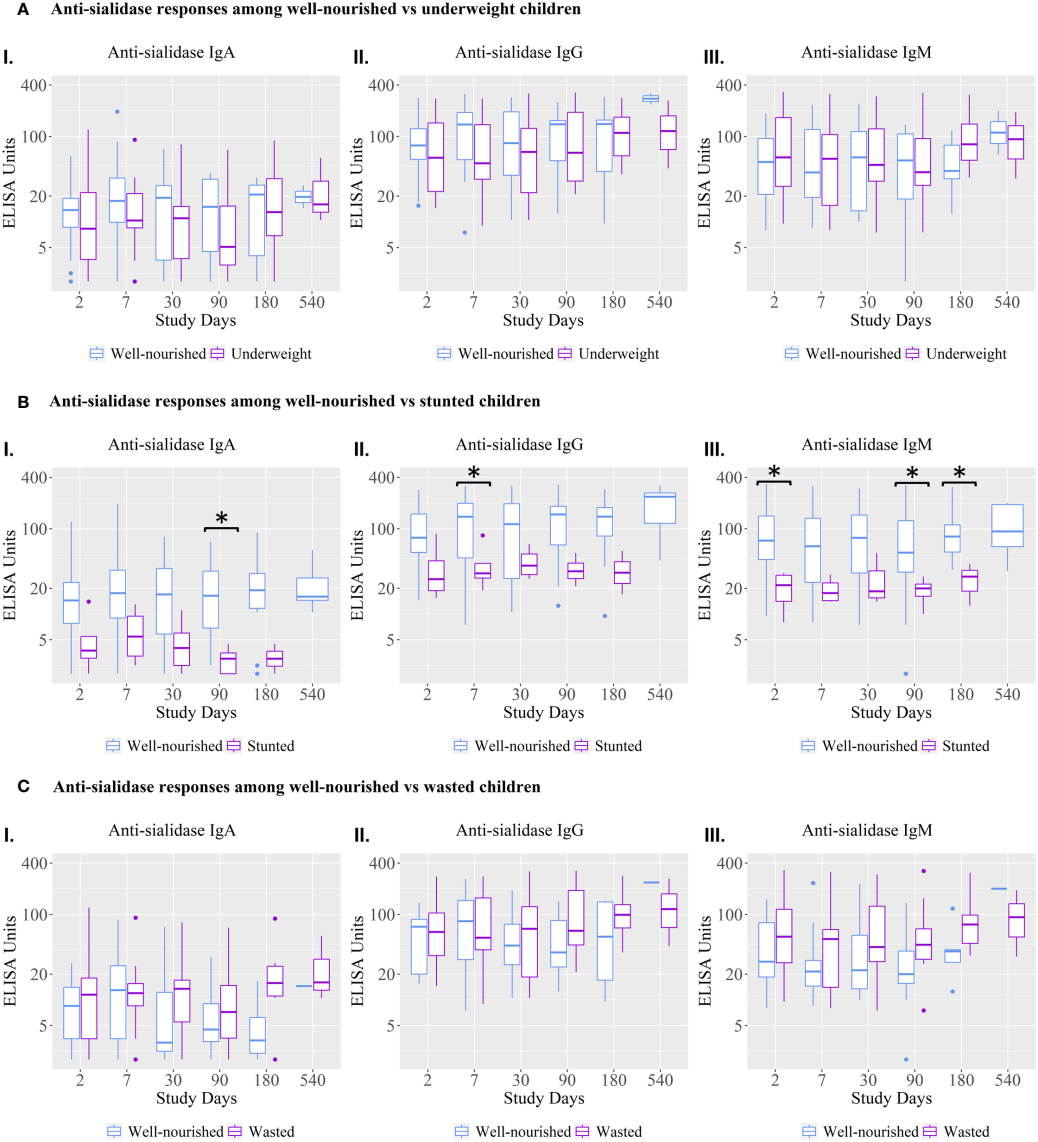
Anti-sialidase IgA, IgG, and IgM antibody responses among well-nourished versus malnourished children. Underweight = weight-for-age; Wasted= weight-for-height; Stunted = height-for-age. Children with a Z score < −2 were categorized as moderately or severely undernourished for that category. Children with a Z score ≥ −2 were categorized as non-moderately or severely malnourished. Kruskal-Wallis test has done in order to identify the significant difference in median responses of anti-sialidase. Asterisks denote the level of significance; *p=0.05- 0.01 and ** denotes p=0.01-0.001. The black asterisks denote the difference between well-nourished and malnourished children.

Anti-sialidase IgA, IgG, and IgM responses were significantly lower at various time points in children who were stunted compared to well-nourished children (**Figure 3**).

**Figure 4:**
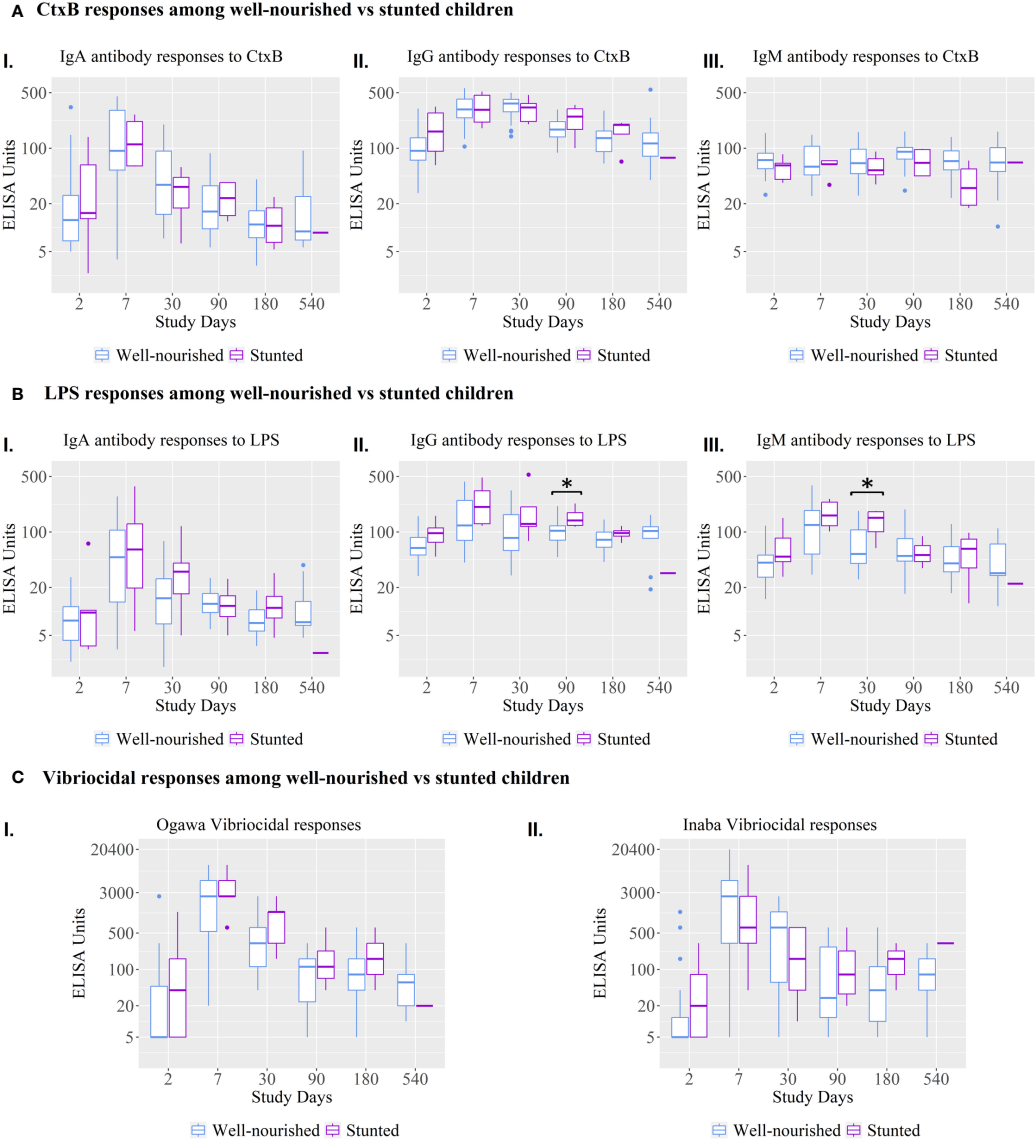
Vibriocidal antibody responses and anti-LPS and anti-CtxB IgA, IgG, and IgM immune responses among well-nourished versus stunted children. Kruskal-Wallis test has done in order to identify the significant difference in median responses of different antibody. Asterisks denote the level of significance; *p=0.05- 0.01. The black asterisks denote the difference between Well-nourished and Stunted Children.

The vibriocidal, anti-LPS IgA and anti-CtxB IgA, IgG, and IgM antibody responses were not statistically significant between stunted and well-nourished children (**Figure 4**), but the stunted children showed significantly higher responses for anti-LPS IgG on day 90 and LPS IgM antibody responses on day 30.

### Anti-sialidase immune responses among cholera patients with concomitant parasitic infection

**Figure 5:**
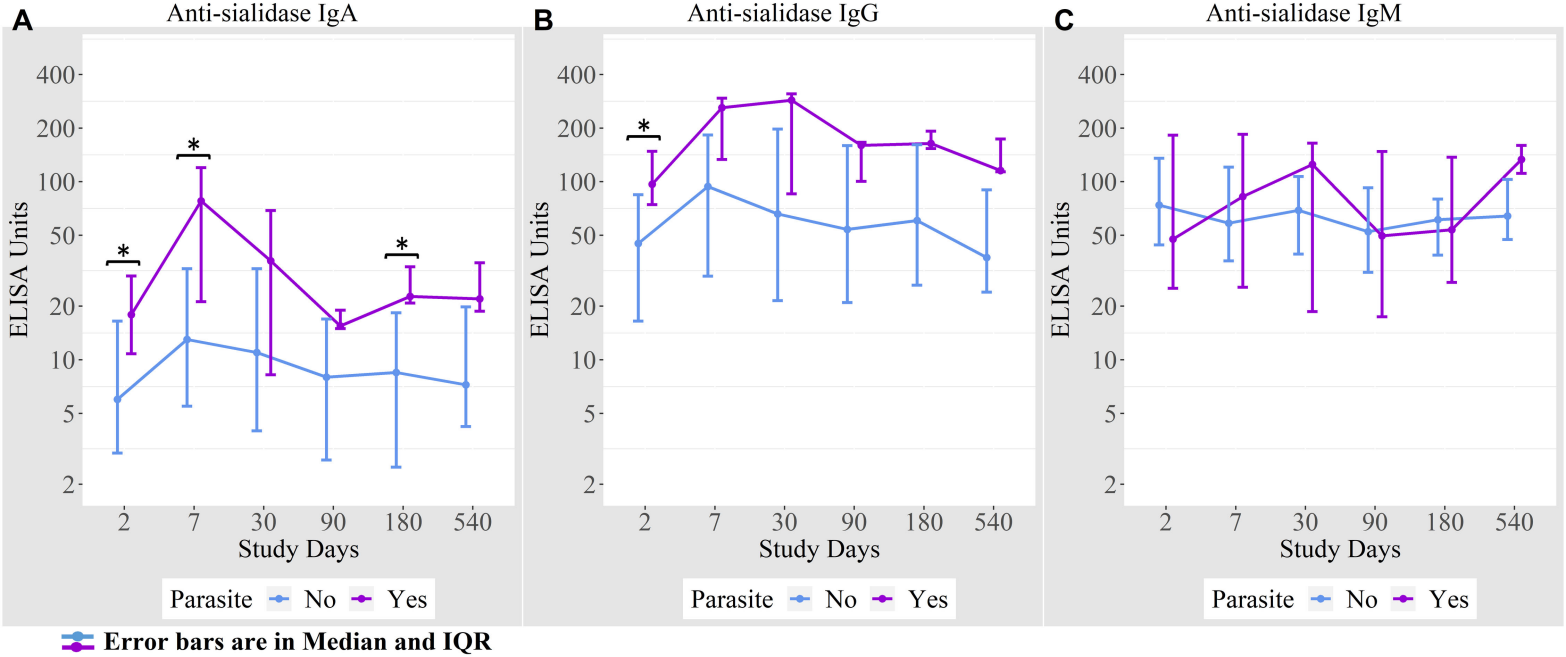
**(A)** Anti-sialidase IgA responses among patients with and without intestinal parasite co-infection **(B)** Anti-sialidase IgG responses among patients with and without intestinal parasite co-infection **(C)** Anti-sialidase IgM responses among patients with and without intestinal parasite co-infection.

We found that 16% of cholera cases were co-infected with intestinal parasites (Ascaris lumbricoides, Trichuris trichiura, Giardia lamblia, and Endolimax nana). Anti-sialidase IgA antibody responses were higher among the cholera cases with a concomitant parasitic infection on days 2, 7, and 180 compared to cholera cases with no parasitic co-infection (**Figure 5**).

We also looked at the anti-sialidase antibody levels in household contacts of the cholera index cases but did not find any significant anti-sialidase antibody responses.

## Discussion

Sialidase is enzyme, acting on sialic acid residues on carbohydrates. Following cholera, patients develop antibody responses to the sialidase produced by *Vibrio cholerae.* Here, we measured anti-sialidase IgA, IgG, and IgM immune responses in children and adults after natural cholera infection out to 540 days following infection and their association with other immunological parameters, nutritional status, and parasitic co-infection. The anti-sialidase IgA and IgG responses were more robust within the first month after infection in adult cholera cases and then gradually decreased from day 30 to day 540. The immediate enhanced anti-sialidase immune responses to *V. cholerae* infection in adults may be associated with a decrease in susceptibility to cholera infection as we have seen in other studies that the immediate rise of vibriocidal and other antibody titers protect individuals from cholera infection (19, 20).

Our previous studies of acute immune responses following *Vibrio cholerae* infection in adults showed that vibriocidal, anti-CtxB, anti-TcpA, anti-LPS, and anti-OSP antibody responses are increased by day 7 after infection; while some of these antibody responses last longer than others, all fall back to baseline levels in the blood within 3-12 months after infection (14, 21, 22). Vibriocidal antibody responses increase sharply by 7 days after infection in symptomatic cases but rapidly wane within 30 days. Anti-CtxB, anti-TcpA, anti-LPS, and anti-OSP antibodies persist for 3-12 months after infection. We find a similar pattern of antibody responses to sialidase in adults in the current study and prior shorter-term study (4), with IgG and IgA responses peaking by day 7 to 30 and then gradually decreasing out to day 540. We have also previously shown that antibodies to CtxB, TcpA, LPS, and OSP provide protection against infection in contacts exposed to cholera in a household if still elevated on re-exposure (19). In a recent study, we showed that anti-sialidase IgG, IgA, and IgM anti-sialidase antibodies are associated with similar protection on household exposure to cholera (4). In addition to differentiation and maturation of B cells to antibody-secreting plasma cells following infection, a subset of B cells differentiates into longer-lasting memory B cells recognizing specific antigens (18, 22, 23). Memory B-cells recognizing CtxB, TcpA, LPS, and OSP develop following cholera and are detectable in circulation from 90 days to 1 year (14, 21, 22). Memory B cell responses to the O antigen of LPS diminish well before one year after infection, while memory B cells recognizing the protein antigens CtxB and TcpA remained elevated to one year and beyond, suggesting that T-cell-dependent antigens may provide longer-lasting memory B cell responses (4). In household contacts exposed to cholera, the level of memory Bcells recognizing CtxB, TcpA, LPS, and OSP antigens detectablein the circulation correlate with protection against subsequentinfection, suggesting that these memory B cells may produce an anamnestic response for longer-term protection. Anti-sialidase-specific memory B cells also develop following infection (1, 4), however, the duration and whether these also correlate with protection on exposure in the household has not yet been evaluated. This would add value to the current seroepidemiological studies based on other immunological markers following *V. cholerae* O1 infection (24).

The anti-sialidase responses were different between children and adults; overall, children lacked significant anti-sialidase immune responses following infection, as also seen in our previous shorter-term study (4). Children, however, do mount robust vibriocidal and LPS and CtxB antibody responses. This discordance can explain the weak to absent associations in children of anti-sialidase responses to other cholera-specific antibody responses (i.e. LPS, CtxB, vibriocidal), in contrast to adults who do mount significant anti-sialidase IgG and IgA and other cholera-specific antibody responses. It is possible that adults develop significant anti-sialidase immune responses after infection because they have been previously exposed to the organism and mount an anamnestic immune response, while children are more likely to be naïve to past infection, and may not mount a significant immune response on primary exposure. Differences in host factors between adults and children, such as malnutrition, enteric enteropathy, intestinal parasite burden, gut microbiome, etc. may also be playing a role (25). The unexpected finding of an increased IgG anti-sialidase response on day 540 in children raised the possibility that children were infected more commonly than adults during the follow-up period, but we considered this unlikely as there were no increases in other immune responses that follow infection, such as vibriocidal or anti-LPS or CtxB responses, in the same children over the same follow-up period. So, we are not certain of the significance or cause of the increased IgG anti-sialidase response seen in children compared to adults on day 540.

During measuring the correlation of antibody responses, we usually correlate the homologous antibody isotypes of antibody-secreting cells (ASC) (26). In this analysis we found some correlation among different antibody isotypes especially in adult patients and this different isotype correlation may have a privileged capacity to generate protective immune responses against cholera; a future study is needed to explain these exceptional findings.

Malnutrition is common in cholera endemic areas and malnutrition is a known factor influencing susceptibility to cholera (8). We found that stunted children (HAZ score <-2) showed significantly lower anti-sialidase IgA, IgG, and IgM antibody responses than well-nourished children; this finding was not seen for children who were malnourished or wasted by WAZ and WHZ scores. This result may be due to our small sample size used to stratify children by “WHO Z Score”. Furthermore, these two markers of malnutrition (WAZ and WHZ scores) represent more recent states of under nutrition than stunting and so their effects may not yet be evident (26). Previous studies have shown that well-nourished children with dehydrating cholera had higher vibriocidal and anti-CtxB responses than adult cases, suggesting that at least well-nourished children can respond satisfactorily to these antigens (27). Very few studies have been conducted to examine immune responses to *V. cholerae* O1 antigens in malnourished children; thus, the complex with different cholera antigens is not yet clear. The study by Falkard et al. found that stunting was associated with an increased response to CtxB (26) but in this study we did not find an increased response to CtxB in stunted compared to well-nourished children, although we did find increased IgM and IgG antibody responses to LPS. It has been reported that LPS-specific antibodies may be higher in LMICs in comparison to the developed world related to gut leakiness (28, 29). This could reflect prior exposure since cholera incidence rates are quite high in young children in LMICs compared to non-endemic-developed settings (20). Environmental enteric dysfunction (EED) in developing countries is a causal factor for stunting (30). A separate cohort of Bangladeshi children from urban slums showed that enteropathy biomarkers specially myeloperoxidase (MPO), and soluble CD14 were positively associated with increased LPS antibody responses among the OCV recipients in children, implying that microbial translocation may augment immune responses to the oral vaccine antigens (31). Moreover, based on the evidence, we may explain that higher LPS antibody responses following cholera in malnourished children may be associated with EED though we did not analyze the biomarkers in stunted children, highlighting the complexity of these interactions.

We also found significantly higher anti-sialidase IgA and IgG responses at various time points in cases of cholera with parasitic co-infection compared to cases of cholera without parasitic co-infection. Immune responses in cholera patients with concomitant parasitic co-infection have not been systematically evaluated. Harris et al. showed that following cholera, IgA immune responses to CtxB were decreased in children with parasitic co-infection, in contrast to other immunological markers like LPS and vibriocidal antibody responses (11). Another study by Araujo et al. analyzed anti-sialidase activity that developed in sera of mice and humans after *Trypanosoma cruzi* parasitic infection, suggesting that the neuraminidase activity of the parasite might play a significant role in the invasion of host cells (32). Here we may explain that neuraminidase in cholera patients infected with parasite plays an augmented role for higher anti-sialidase immune response. Moreover, parasites modulate the host immune response, especially T cell-dependent pathways (33, 34), and its effects on LPS antibody levels may not be similarly impacted as the LPS is a T cell-independent antigen (11).

This study has some limitations. We included relatively small sample size and did not analyze stool samples for mucosal immune responses and excluded demonstration of antibody functionality. Furthermore, we did not evaluate the duration of memory B-cell or development of T-cell responses against sialidase among study participants, and did not evaluate whether long-term anti-sialidase antibody responses were induced by currently available oral cholera vaccines. No healthy participant was included in this analysis to compare the baseline response. Future study related to these issues may show new insight to more knowledge regarding anti-sialidase responses.

Notwithstanding these limitations, we have conducted a longitudinal cohort study in an endemic setting for cholera and followed anti-sialidase antibody responses in adults and children over an extended period of time. This cohort compared the immune responses in stunted vs well-nourished children and also studied of the cholera cases who were co-infected with intestinal parasites.

Future studies are needed to examine anti-sialidase memory B and T cell development and duration following natural infection and vaccination, as well as the role these cells may play in protection from subsequent infection.

## Data availability statement

The original contributions presented in the study are included in the article/supplementary material. Further inquiries can be directed to the corresponding author.

## Ethics statement

This study was reviewed and approved by the Institutional Review Board, icddr,b. Written informed consent was taken to participate in this study, which was provided by the participant or participant's legal guardian/next of kin.

## Author contributions

FC, FQ, and TB designed and supervised the study. FC, TB, AA, IT, and RB helped to collect the specimens, performed the laboratory work and immunological analyses. FC, AA, TB, and MF analyzed the data. FQ, FC, RC, and ER provided key reagents. FC, and AA drafted the manuscript. FQ, FC, SC, AA, TB, RB, JH, RL, AGR, NM, RC, ER and IT reviewed the manuscript. All authors contributed to the article and approved the submitted version.
